# Conflicting Conclusions or Competing Methodologies? Documenting Soil Lead Pollution in Owino Uhuru, Kenya

**DOI:** 10.5696/2156-9614-9.21.190301

**Published:** 2019-03-13

**Authors:** Jack Caravanos

**Affiliations:** Editor in Chief, Journal of Health and Pollution, Clinical Professor of Environmental Public Health Sciences, College of Global Public Health, New York University

This issue of the *Journal of Health and Pollution* (*JH&P*) presents a follow-up paper submitted by Ericson et al.^2^ on the lead contamination and poisoning situation in the neighborhood of Owino Uhuru in Mombasa County, Kenya. This controversial and politically charged community environmental contamination battle is ongoing, with complex lead exposure pathways still not properly defined.

In June 2018, this journal published the work of Etiang et al.^1^ The authors took childhood blood lead level (BLL) measurements (n=130) as well as limited samples of lead in soil (n=8), dust (n=7) and drinking water (n=15) from 130 households. They concluded that, among other activities, “*The high lead level of soil in Owino Uhuru (neighborhood) illustrates the need for coordinated efforts to remediate the environment*.”^1^ They furthermore stated that “*This requires removal of the first several inches of soil and replacing with clean fill, which may necessitate the removal of children from their homes until the environment is confirmed to be safe*.”^1^

Meanwhile, Ericson's paper published in the present issue concludes that “*The need for mitigation work in the Owino Uhuru may not be as pressing as presented elsewhere*.”^2^

This editorial therefore addresses an important question-how can researchers studying the same condition come to such seemingly different conclusions? How are journals, reviewers and editorial boards to cope with such discrepancies? To put it directly, new data often become available that may supersede previously published findings. The ethical and professional practice of journals and reviewers should be to present this new information, despite an apparent challenge to earlier findings.

However, these papers, while related, are each distinctly different in their methodologies. Ericson et al. conducted extensive community lead in soil profiling, while Etiang et al. did not.^1^,^2^ Meanwhile, Etiang et al. conducted actual testing of children's blood in both the “exposed” and “unexposed” neighborhoods, while Ericson relied on the United States Environmental Protection Agency's “Integrated Exposure, Uptake and Biokinetic Model” to predict children's BLLs.^1^,^2^

Ericson et al. further used established lead deposition models in an attempt to predict Etiang et al.'s observed lead in soil observation, with little success.^1^,^2^

What we do know from assimilating the information in these two papers is that there is a historic lead smelter in Owino Uhuru that likely contributed to the elevated BLLs observed by Etiang et al.^1^ Additionally, we know that levels of lead in soil vary significantly in these communities and it appears it is entering residential dwellings. What still needs to be determined is the precise exposure source and pathway. However, as Ericson points out “*… the burden of proof to justify interventions should be high*,” especially since “*The residents of Owino Uhuru are subject to myriad environmental health and livelihood risks common to life in informal settlements*”.^2^ Blood lead levels, soil concentrations and residential lead dust levels reported in 2015 may not apply to conditions in 2017 or even to the present day. Before limited vital resources are directed to address childhood lead poisoning in Owino Uhuru, a clear and scientifically defensible environmental exposure pathway must be identified. Perhaps we will see yet another paper clarifying this question in the years to come.
